# Supplementary material for: The genome sequence of the Mountain Dappled White, Euchloe simplonia (Boisduval, 1828) (Lepidoptera: Pieridae)
Source: Wellcome Open Res. 2025 Aug 15;10:446. [Version 1] doi: 10.12688/wellcomeopenres.24695.1 (PMC12586986; doi:10.12688/wellcomeopenres.24695.1)
Supplement: Peer review history

# Reviewer report: Reviewer response for version 1

**DOI:** 10.21956/wellcomeopenres.27211.r135728

**Published:** 2025-11-04

**Authors:** Panagiotis Ioannidis

**Affiliations:** 1 Foundation for Research & Technology - Hellas, Crete, Greece

This paper describes the sequencing and assembly of the lepidopteran *Euchloe simplonia*. The authors provide adequate information about the ecology of the species in the Introduction. The methodology used is the standard one, used by other DToL papers. 

 The genome assembly metrics are exceptional; complete BUSCOs: 98.9%, QV = 65, k-mer completeness = 99.64%, and CC ratio = 76/31 = 2.45. As such, this genome assembly is suitable for downstream analyses. 

 As I have mentioned in other recent reviews of mine, I believe that the usefulness of genome assemblies without the accompanying predicted gene sets (i.e. genome annotations) is limited. Especially for people with limited skills in bioinformatics such as wet-lab biologists who want to study a particular gene or gene family.

Are sufficient details of methods and materials provided to allow replication by others?

Yes

Is the rationale for creating the dataset(s) clearly described?

Yes

Are the datasets clearly presented in a useable and accessible format?

Yes

Are the protocols appropriate and is the work technically sound?

Yes

Reviewer Expertise:

bioinformatics; insect genomics

I confirm that I have read this submission and believe that I have an appropriate level of expertise to confirm that it is of an acceptable scientific standard.

# Reviewer report: Reviewer response for version 1

**DOI:** 10.21956/wellcomeopenres.27211.r135726

**Published:** 2025-10-30

**Authors:** Matthew Merkin

**Affiliations:** 1 University of Liverpool, Liverpool, UK

Summary:

 The authors present a new chromosome level assembly of a European butterfly, the Mountain Dappled White ( *Euchloe simplonia*), as part of Project Psyche. DNA was extracted and sequenced from the head (HiC) and thorax (HiFi) of a single individual, followed by assembly, scaffolding and manual curation. The quality of the final assembly is high (BUSCO C:98.9%), suggesting it is suitable for use as a reference genome. The mitochondrial genome was also assembled. 

 The inclusion of Table 1 (sequencing information) and Figure 3 (assignment of Merian elements) greatly improves this genome note by allowing the reader to more easily access sequencing data and identify equivalent chromosomes across species.

 Minor comments:

 -There is little rationale for why this species was sequenced. An additional sentence expanding on Project Psyche and its goal to sequence lepidopterans would be useful here, especially if it can be related to this sentence from the roadmap on their website “ *We will prioritise sequencing for phylogenetic diversity across the order and deliver genomes for agricultural pests, ecological indicator species and threatened species.*” - this could tie in to your speculation that this species is endangered in some areas, or just that there are comparatively few Pierid genomes.

 -Is there a reason why a male sample was used or no annotation was produced? The W chromosome and protein information provided in the DToL genome notes was highly useful. I see there is a commitment in the Data Availability section to utilise RNA-seq data later on, in which case the publication of this genome note seems premature. 

 -The inclusion of benchmark values in Table 4 is highly useful for statistics that are unfamiliar to many, such as EBP summary. However, it can be hard to compare the value and benchmark in three rows: 

 1) The EBP summary in the table (6.7.Q65) differs from the main text (6.C.Q65) in the central scaffold NG50 field. Although the values are explained briefly in the linked articles, it may also be worth including an additional sentence explaining them to an unfamiliar reader.

 2) The benchmark for the scaffold N50 is “= chromosome N50”. It would help to list the actual value of the chromosome N50 here as well.

 3) “Percentage of assembly assigned to chromosomes” has a value of 0%, which does not seem correct. The sum of chromosome lengths in Table 3 is 395.34, which when divided by the assembly span in Table 2 (397.32), and multiplied by 100 gives a value of ~99.5%.

Are sufficient details of methods and materials provided to allow replication by others?

Yes

Is the rationale for creating the dataset(s) clearly described?

No

Are the datasets clearly presented in a useable and accessible format?

Yes

Are the protocols appropriate and is the work technically sound?

Yes

Reviewer Expertise:

Comparative genomics, genetics, entomology, ancient DNA, population genetics

I confirm that I have read this submission and believe that I have an appropriate level of expertise to confirm that it is of an acceptable scientific standard.

# Reviewer report: Reviewer response for version 1

**DOI:** 10.21956/wellcomeopenres.27211.r131315

**Published:** 2025-10-08

**Authors:** Christopher B. Cunningham

**Affiliations:** 1 University of Georgia, Georgia, USA

The project continues to produce quality resources for the insect genomics community. The project for the Mountain Dappled White, *Euchloe simplonia*, continue that. The methods are sound and relatively well documented. The article needs has enough details to be reasonably reproducible with the newer addition of pipeline documentation. The data supporting the publication are publicly available.

 Abstract. There is no justification within the abstract; just results. At least a sentence that this was part of a large survey of insects is needed. And one sentence for its possible value as a member of an under sampled group or ecology or something to motivate reader interest.

 Keywords. There are all repeated from the title or abstract. They have little value to the reader. Keywords are used by Google to index the work so should reflect its broader use.

 Background. Background. The stated motivation for this study in the introduction is fine – part of a very large survey. However, it would be nice for the reader to understand if this is the first of its genus, is of particular interest for ecological or genetics studies, or something unusual about its biology. Helping the reader understand what this genome might help with investigation in. Genus names at the beginning of sentences should be spelled out.

 Results. Table 1 is not really needed. They are not details that the reader needs to under the work and they can all be found under the NCBI BioProject ID or are repeated from the methods paragraph.

 BUSCO completeness needs to be assessed with the updated OrthoDB version 12. Difference from 10 to 12 have made a big difference for several insects I work with and colleagues have reported the same. The paper should not be indexed until this is updated. The new version has been for 11 months.

 Figure 4. Fig 4 has very little information beyond what is found elsewhere in the manuscript and can easily be summarized as a sentence in the results.

 Figure 3 & 5. Suggest lots of assembly dead-ends. I appreciate that they have no hits to anything other than Arthropoda but still the variation in coverage, variation in GC content, and strong Hi-C links among themselves is very odd. It suggests the QC of the genome draft assembly might be incomplete.

 Genome annotation is not presented. This greatly limits the use of this resource.

Are sufficient details of methods and materials provided to allow replication by others?

Yes

Is the rationale for creating the dataset(s) clearly described?

No

Are the datasets clearly presented in a useable and accessible format?

Yes

Are the protocols appropriate and is the work technically sound?

Yes

Reviewer Expertise:

Insects, genetics, genomics, epigenetics, behavior, reproduction.

I confirm that I have read this submission and believe that I have an appropriate level of expertise to confirm that it is of an acceptable scientific standard, however I have significant reservations, as outlined above.
